# Myeloid Cell Leukemia-1 knockout leads to increased viral propagation of Respiratory Syncytial Virus and influenza virus in mouse embryonic fibroblast cells and A549 cells: implications in cancer therapy

**DOI:** 10.3389/fcimb.2025.1615790

**Published:** 2025-08-29

**Authors:** Meagan A. Prescott, Maciej Maselko, Manoj K. Pastey

**Affiliations:** ^1^ Department of Biomedical Sciences, Carlson College of Veterinary Medicine, Oregon State University, Corvallis, OR, United States; ^2^ Department of Microbiology, College of Science, Oregon State University, Corvallis, OR, United States; ^3^ Molecular and Cellular Biology Program, Oregon State University, Corvallis, OR, United States

**Keywords:** Respiratory Syncytial Virus, influenza virus, Myeloid Cell Leukemia-1, Mcl-1 inhibitor therapies, small interfering RNA

## Abstract

**Introduction:**

Respiratory Syncytial Virus (RSV) remains a significant global health burden, particularly affecting young children, elderly individuals, and immunocompromised patients. The antiapoptotic protein Myeloid Cell Leukemia-1 (Mcl-1) is rapidly upregulated following RSV infection; however, its functional significance in viral pathogenesis remains poorly defined.

**Methods:**

We investigated the role of Mcl-1 during RSV infection using Mcl-1 knockout mouse embryonic fibroblasts (ΔMcl-1 MEFs) and human alveolar epithelial (A549) cells subjected to small interfering RNA (siRNA)-mediated Mcl-1 knockdown. Viral replication was quantified by plaque assays, and phenotypic effects were assessed through syncytia formation and apoptosis assays. To assess broader implications, influenza A virus replication was also evaluated in ΔMcl-1 MEFs and Mcl-1–silenced A549 cells.

**Results:**

RSV replication was significantly enhanced in ΔMcl-1 MEFs compared to wild-type (WT) controls, with increased viral titers, larger syncytia formation, and elevated apoptosis during the late stages of infection. Consistent results were observed in A549 cells following Mcl-1 knockdown, where RSV titers increased by more than 3 log₁₀. Influenza A virus replication was also markedly elevated in ΔMcl-1 MEFs and siRNA-treated A549 cells, suggesting that Mcl-1 exerts a broad antiviral effect across multiple respiratory viruses.

**Discussion:**

These findings indicate that Mcl-1 upregulation during RSV and influenza virus infection functions as a critical host antiviral defense mechanism, rather than a viral evasion strategy. Clinically, our results raise concerns regarding therapies that target Mcl-1, such as certain anticancer treatments, which may inadvertently increase susceptibility to severe viral infections. Careful monitoring and potential prophylactic antiviral interventions may be warranted in patients receiving Mcl-1 inhibitor therapies.

## Introduction

Acute respiratory infections are a major contributor to the global health burden. Human Respiratory Syncytial Virus (RSV) is a leading cause of respiratory infection across all age groups. It predominantly causes severe disease in young children, the elderly, and those with compromised immune systems (Bem, 2023; Branche and Falsey, 2015; He et al., 2025; Langley and Anderson, 2011; Murata and Falsey, 2007; Nair et al., 2010; Walsh and Falsey, 2012; Verani et al., 2013; Lee et al., 2013). The annual incidence of RSV infection in children under five is estimated at nearly 40 million cases globally, and by two years of age nearly all children in the U.S. have been infected at least once (Nair et al., 2010; Ogra, 2004; Borchers et al., 2013).

RSV is an enveloped negative-sense RNA virus of the *Pneumoviridae* family (Afonso et al., 2016). Several RSV proteins, including the nonstructural proteins NS1 and NS2 and the envelope fusion (F) and small hydrophobic (SH) proteins, have been shown to influence apoptotic processes during infection (Li et al., 2015; Eckardt-Michel et al., 2008; Bitko et al., 2007).

Apoptosis is a critical aspect of host–virus interactions, and many viruses either suppress or induce cell death to enhance their replication and spread (McLean et al., 2008). The BCL-2 family of proteins tightly regulates apoptosis, consisting of pro-apoptotic effectors (e.g., BAX, BAK, BOK, BID, BIM, BAD, NOXA, PUMA) and anti-apoptotic members (e.g., BCL-2, BCL-W, BCL-XL, A1, and Mcl-1) (Youle and Strasser, 2008). Evasion of apoptosis is a common strategy in viral infection: many viruses encode anti-apoptotic factors to delay or prevent host cell death, while others actively trigger apoptotic pathways at specific stages to facilitate their dissemination (Liang et al., 2015; Ghosh Roy et al., 2014; Krejbich-Trotot et al., 2011). For instance, blocking Mcl-1 during the entry stage inhibits SARS-CoV-1 infection by preventing membrane fusion (Mao et al., 2022). Some viruses also encode pro-apoptotic proteins to promote host cell death under certain conditions. Notably, the SARS-CoV-1 7a protein induces apoptosis by binding the host anti-apoptotic protein BCL-XL (Tan et al., 2007). African swine fever virus (ASFV) encodes both pro- and anti-apoptotic proteins (e.g., pE183L and pA179L, respectively), suggesting that ASFV modulates apoptosis differently at various stages of its replication cycle (Li et al., 2021).

An example of the diverse ways viruses manipulate apoptosis is the tumor suppressor p53. This key positive regulator of apoptosis is targeted for degradation by adenovirus and papillomavirus and is inhibited by Kaposi’s sarcoma-associated herpesvirus. In contrast, p53 is *activated* during infections by Rift Valley fever virus, HIV, influenza virus, and West Nile virus, leading to apoptosis (Austin et al., 2012; Chudasama et al., 2015; Huibregtse et al., 1991; Steegenga et al., 1998; Suo et al., 2015; Verma et al., 2011; Wang et al., 2014; Yang et al., 2008; Eckardt-Michel et al., 2008). RSV’s modulation of apoptosis is complex, and findings have been somewhat conflicting. Early during RSV infection, apoptosis is suppressed by multiple mechanisms – including p53 degradation, delayed activation of EGFR, and upregulation of anti-apoptotic host factors like NF-κB – to prolong cell survival (Groskreutz et al., 2007; Bitko et al., 2007; Thomas et al., 2002; Lindemans et al., 2006; Monick et al., 2005). However, at later stages of RSV infection, apoptosis is actively induced via pathways involving TRAIL, FasR, ER stress-activated caspase-12, inducible nitric oxide synthase (iNOS/NO), and p53-triggered mechanisms via the RSV F protein (Bem et al., 2010; Bitko and Barik, 2001; Mgbemena et al., 2013; Eckardt-Michel et al., 2008; Kotelkin et al., 2003; O’Donnell et al., 1999). In summary, RSV dynamically balances apoptosis suppression and induction at different times during infection, and the full details of this balance remain incompletely understood.

**Figure 1:**
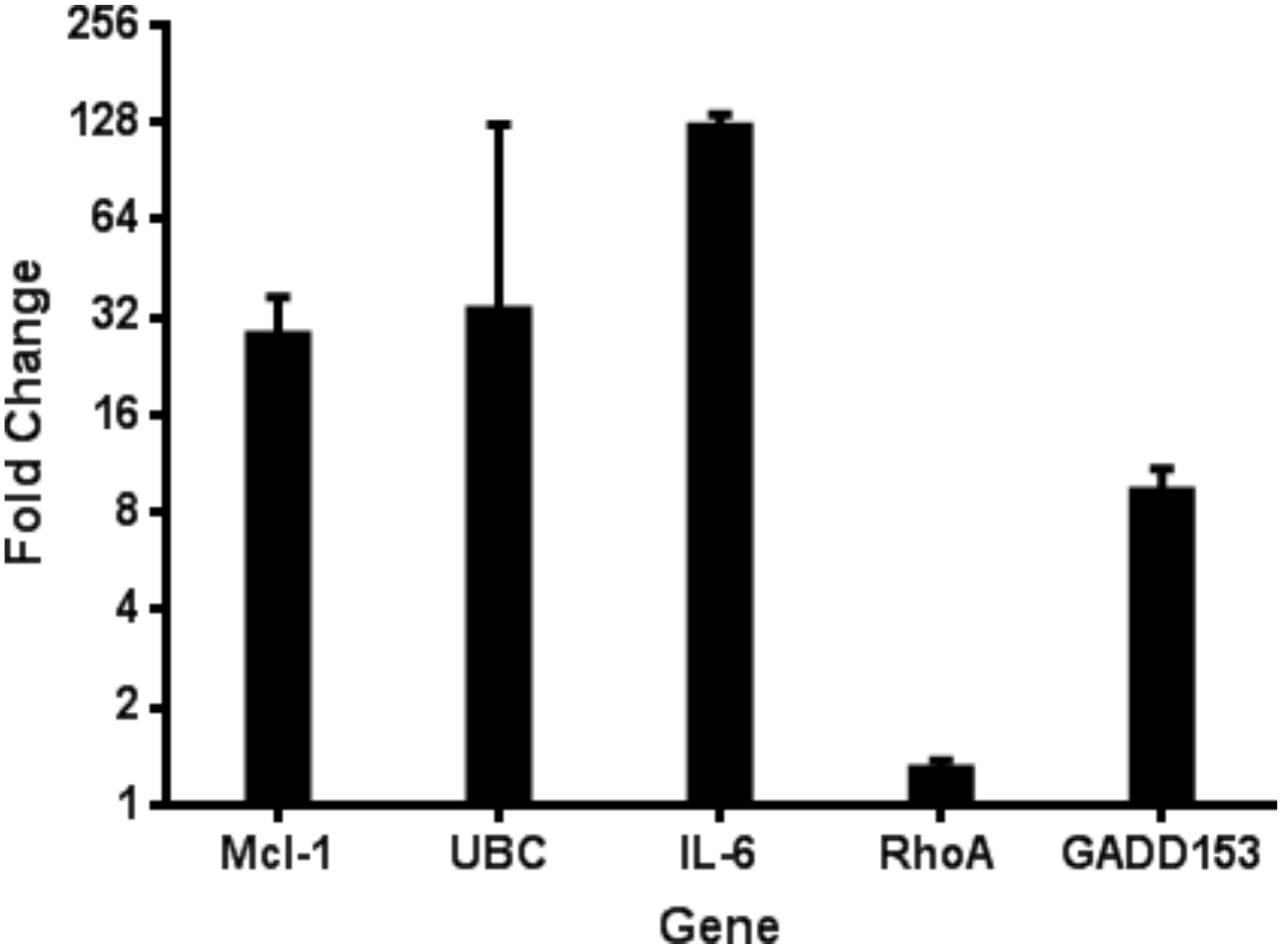
Quantitative RT-PCR verification of mRNA levels for several host genes during RSV infection. Microarray analysis of host gene expression during RSV infection indicated several upregulated host genes. The upregulation of these genes was confirmed using the Invitrogen SuperScript III Platinum SYBR Green One-step qRT-PCR kit from HEp-2 cells 24 hpi with RSV. Data is represented as GAPDH normalized fold change of RSV infected cells compared to uninfected controls.

Myeloid Cell Leukemia-1 (Mcl-1) is an anti-apoptotic BCL-2 family protein that is notably upregulated in RSV infection after initial infection (**Figure 1**). Mcl-1 performs its anti-apoptotic function at the outer mitochondrial membrane by sequestering pro-apoptotic effectors BAX and BAK (Perciavalle et al., 2012). It remains unclear whether Mcl-1’s upregulation during RSV infection benefits the virus or the host. Here, we address this question by comparing RSV infection in WT and Mcl-1–knockout MEF cells. We demonstrate that MEFs lacking Mcl-1 replicate RSV and influenza virus much more efficiently than WT MEFs. We also describe distinct morphological changes (especially in syncytium formation) in ΔMcl-1 MEFs upon RSV infection and investigate whether these effects are linked to apoptosis. Mirroring our findings in the mouse fibroblast system, the absence or reduction of Mcl-1 greatly enhances RSV and influenza virus replication even in human lung cells (A549). Importantly, this raises a potential concern: patients receiving Mcl-1 inhibitor drugs (as part of anti-cancer therapy) might become more susceptible to severe RSV and influenza virus infection or other respiratory viral diseases, due to the virus being able to replicate uncontrolled when Mcl-1 is suppressed. Our findings also suggest that Mcl-1 has a functional role in RSV infection beyond its classical anti-apoptotic activity.

## Materials and methods

### Cells and virus

MEF cells (wild-type and ΔMcl-1 knockout) were kindly provided by Dr. Joseph T. Opferman (St. Jude Children’s Research Hospital, Memphis, TN, USA). Generation of the ΔMcl-1 MEF line was previously described (Opferman et al., 2003). Murine RAW 264.7 macrophage cells were provided by the Bermudez Laboratory (Oregon State University, Corvallis, OR, USA). HEp-2, HeLa, A549, and MDCK cell lines were obtained from the American Type Culture Collection (ATCC, Manassas, VA, USA). All cells were maintained in Dulbecco’s Modified Eagle Medium (DMEM) supplemented with 10% fetal bovine serum (FBS) at 37 °C in a 5% CO_2_ atmosphere.

RSV strain VR-1540 (subtype A2, ATCC) was used for all RSV experiments. Viral stocks were prepared following a published protocol (Ueba, 1978) with minor modifications. Briefly, HEp-2 cells were infected with RSV at a multiplicity of infection (MOI) of 0.5 and incubated for two days. A single plaque was then isolated and used to infect a fresh T-75 flask of HEp-2 cells. After an additional three-day incubation, cells and supernatant were collected and vigorously vortexed, and cells were pelleted by centrifugation (10 minutes at 3,000 × *g*, room temperature). The supernatant was layered onto a 30% sucrose solution (30% sucrose w/v in 1 M MgSO_4_, 50 mM HEPES, 150 mM NaCl) and ultracentrifuged at ~60,000 × *g* for 30 minutes at 4 °C. The resulting virus pellet was collected and centrifuged once more to further concentrate and purify the virus. Viral aliquots were snap-frozen in a dry ice–ethanol bath and stored at –80 °C. Viral stock titers were determined periodically by plaque assay.

For influenza virus experiments, influenza A/PR/8/34 (H1N1) was used. Stock H1N1 virus was grown in embryonated chicken eggs using standard techniques and stored in aliquots at –80 °C until use (Brauer and Chen, 2015; Balish et al., 2013).

### cDNA microarray analysis

We analyzed differential gene expression in HEp-2 cells infected with RSV using cDNA microarray analysis complemented by quantitative PCR (qPCR). Briefly, cells were harvested at 24 hours post-infection (hpi), and total cellular RNA was extracted using the RNeasy Mini Kit (Qiagen) with on-column DNase I digestion to eliminate genomic DNA. RNA quality was confirmed by UV spectrophotometry and agarose gel electrophoresis. Radiolabeled cDNA probes were synthesized from 2 μg total RNA per sample using the Atlas^®^ Pure Total RNA Labeling System (Clontech) with ^32^P labeling. Probes from RSV-infected and mock-infected samples were hybridized separately overnight at 68 °C to Atlas^®^ Human Cancer cDNA Expression Arrays (Clontech), containing 588 duplicated cDNA targets. Membranes were washed under high-stringency conditions and exposed to Kodak BioMax MS autoradiography film for 24 hours at –70 °C. Signal intensities were quantified using ImageJ software, and gene expression was calculated as the fold-change ratio (RSV/mock) after global normalization excluding external controls. Statistical analysis was performed on the data derived from at least three independent experiments to determine significant changes in gene expression. Additionally, to validate the microarray findings, we conducted **quantitative real-time PCR (qPCR)** on a subset of the differentially expressed genes. cDNA was prepared from independent RNA isolates of mock and RSV-infected HEp-2 cells (24 hpi), and qPCR was performed using gene-specific primers (designed for GADD153, IL6, MCL1, UBC, and others) designed using NCBI Primer-BLAST, with SYBR Green detection on an ABI Prism system. Expression levels were normalized to an internal reference (18S rRNA) and calculated by the delta-delta CT (ΔΔC_t) method. The qPCR results were compared to the microarray fold-change data for consistency.

### Plaque assay for RSV

RSV titers were determined by plaque assay on >90% confluent HeLa cell monolayers. Samples (cell supernatants) were serially diluted in culture medium, and each dilution was added to triplicate wells of HeLa cells. After adsorption for one hour at 37 °C, the inoculum was removed, and the cells were overlaid with medium containing 1% low-melting-point agarose in DMEM + 10% FBS. Once the overlay solidified, plates were inverted and incubated at 37 °C for four days. Cells were then fixed with 4% paraformaldehyde for two hours, and the agarose plugs were removed. Plaques were visualized by immunostaining: monolayers were blocked with 5% non-fat dry milk in PBS, then incubated with primary goat anti-RSV polyclonal antibody (Millipore AB1128, Billerica, MA, USA) at an appropriate dilution. After three washes with PBS + 0.05% Tween-20, cells were incubated with an HRP-conjugated donkey anti-goat secondary antibody (Rockland Immunochemicals, Pottstown, PA, USA) and washed again. Plaques were developed using the chromogenic substrate TMB (3,3’,5,5’-tetramethylbenzidine) (BioFX, Owings Mills, MD, USA) and then counted. Titers were calculated as plaque-forming units per milliliter (PFU/mL). All RSV plaque assay experiments were independently repeated three times, each with triplicate wells, and mean values are shown.

### Plaque assay for influenza virus

Influenza virus titers were measured by plaque assay on MDCK cells. MDCK monolayers (~90% confluent) were inoculated with serial dilutions of virus and incubated for one hour at 37 °C. After removing the inoculum and washing with PBS, cells were overlaid with Eagle’s Minimum Essential Medium (EMEM) containing 7.5% bovine serum albumin, antibiotics, 2 mM glutamine, 1.2 µg/mL TPCK-trypsin, and 1% low-melt agarose. After the overlay solidified, plates were inverted and incubated at 37 °C for five days. Cells were then fixed with 4% paraformaldehyde for two hours, the agarose plugs were removed, and the monolayers were stained with 0.5% crystal violet in water for 30 minutes. Excess stain was rinsed off, plates were dried, and plaques were counted to determine PFU/mL All influenza plaque assay experiments were independently repeated three times, each with triplicate wells, and mean values are shown.

### Replication experiments

Viral replication kinetics in the MEF cell lines were assessed by multi-step growth curve experiments. In all cases, MEFs were seeded one day prior to infection. Cells were infected with RSV or influenza at the specified MOI for each experiment and incubated for the indicated times. At each time point, cell culture supernatants were collected and titrated by the appropriate plaque assay method described above to quantify viral replication. All virus replication experiments were independently repeated three times, each with triplicate wells, and mean values are shown.

### Apoptosis assay

Caspase-3/7 activity was measured to assess apoptosis in RSV-infected MEFs. One day before infection, wild-type (WT) and Mcl-1 knockout MEFs (ΔMcl-1 MEFs) were seeded into a clear-bottom 96-well plate (PerkinElmer). Cells were infected with RSV at an MOI of 2 or mock-infected with medium (in triplicate for each condition). At 12, 24, 36, and 48 hours post-infection (hpi), caspase activity in parallel sets of wells was measured using the Apo-ONE Homogeneous Caspase-3/7 assay (Promega) according to the manufacturer’s instructions. This assay uses a pro-fluorescent caspase substrate that, when cleaved by active caspase-3/7, produces a fluorescent rhodamine 110 signal. Fluorescence was measured at 485 nm excitation/535 nm emission using a Tecan F200 plate reader (Tecan Group Ltd, Männedorf, Switzerland). To normalize for cell number and baseline apoptosis, the fluorescence reading from each infected well was divided by the average reading of mock-infected wells of the same cell type at the corresponding time point. These normalized ratios (infected vs. mock) were used to compare caspase activity between WT and ΔMcl-1 cells over time.

### Confocal microscopy

To visualize RSV infection, MEF cells were grown on sterile glass coverslips (pre-coated with poly-L-lysine) and infected with RSV at an MOI of 2. After one hour of virus adsorption, the inoculum was removed and replaced with fresh culture medium. At designated time points (12, 24, 48, and 72 hpi), coverslips were washed with cold PBS and cells were fixed with 4% paraformaldehyde. Fixed cells were then permeabilized with 0.2% Triton X-100 in PBS for 5 minutes and washed with PBS. Cells were blocked with 2% BSA in PBS for 30 minutes, followed by staining with primary goat anti-RSV polyclonal antibody (EMD Millipore) at 1:100 dilution for one hour. After washing, cells were incubated with Alexa Fluor 488–conjugated rabbit anti-goat secondary antibody (Life Technologies) at 1:200 for 45 minutes. Nuclei were counterstained with DAPI, and coverslips were mounted onto slides using Vectashield mounting medium (Vector Labs). Imaging was performed with a Zeiss LSM 510 META confocal microscope (63× oil-immersion objective). For controls, infected cells stained with secondary antibody only and uninfected cells with full staining protocol were prepared to check for nonspecific staining. At least 30 fields of view per sample were examined, and representative micrographs were captured.

### Western blots

Protein lysates were collected to verify Mcl-1 expression levels in various cells. Wild-type (WT) and Mcl-1 knockout MEFs (ΔMcl-1 MEFs), as well as control human A549 and murine RAW 264.7 cells, were lysed in RIPA buffer (Thermo Scientific) containing a protease inhibitor cocktail (G-Biosciences). Lysates were mixed with NuPAGE LDS sample buffer and 500 µM dithiothreitol (DTT), then heated at 70 °C for 10 minutes. Equal volumes of each sample were loaded onto a NuPAGE Bis-Tris gel (Invitrogen) alongside molecular weight markers and electrophoresed for ~40 minutes at 200 V. Proteins were transferred onto a nitrocellulose membrane using an XCell II Blot Module (Invitrogen) semi-wet transfer apparatus for 90 minutes at 30 V. Membranes were blocked overnight at 4 °C in near-infrared blocking buffer (Rockland). Blots were first probed with a mouse anti-β-actin primary antibody (Santa Cruz Biotechnology) as a loading control, then washed and probed with a rabbit anti-Mcl-1 primary antibody (Cell Signaling D35A5) which recognizes both human and mouse Mcl-1. After washing, blots were incubated with IRDye 800 conjugated anti-rabbit IgG and IRDye 680RD anti-mouse IgG secondary antibodies (Rockland). Bands were visualized using a Li-COR Odyssey infrared imaging system.

### Apoptosis induction in WT MEFs by Camptothecin

To test the effect of inducing apoptosis on RSV replication, wild-type (WT) MEFs were treated with Camptothecin (CPT) (R&D Systems, Minneapolis, MN, USA), a pro-apoptotic drug, during infection. WT MEFs were seeded in triplicate wells and infected with RSV at MOI 2. After the 1-hour adsorption at 37 °C, the inoculum was removed and replaced with fresh medium containing either CPT or an equivalent volume of solvent (DMSO). Two concentrations of CPT were used: 2 µM (with 0.1% DMSO) and 4 µM (with 0.2% DMSO), with DMSO-only control wells containing the same final DMSO percentages. The virus was allowed to replicate for 24 hours. Supernatants were then collected and RSV titers in each condition were measured by plaque assay.

### Apoptosis inhibition in Mcl-1 knockout MEFs by Z-VAD-FMK

To assess the effect of blocking apoptosis on RSV replication, Mcl-1 knockout MEFs (ΔMcl-1 MEFs) were treated with the pan-caspase inhibitor Z-VAD-FMK (R&D Systems, Minneapolis, MN, USA) during infection. ΔMcl-1 MEFs were seeded in triplicate wells and infected with RSV at MOI 2 for one hour. After removing the inoculum, fresh medium containing either 100 µM Z-VAD-FMK or 0.5% DMSO (vehicle control) was added to the respective wells. Virus was allowed to replicate for 24 hours, and supernatants were collected for plaque assay titration of RSV.

### small interfering RNA-mediated knockdown of Mcl-1 in human alveolar epithelial (A549) cells

Human type II alveolar epithelial (A549) cells were obtained from the American Type Culture Collection (ATCC, Manassas, VA, USA) and cultured in Dulbecco’s Modified Eagle Medium (DMEM) supplemented with 10% fetal bovine serum (FBS) at 37 °C in a humidified incubator with 5% CO_2_. For small interfering RNA (siRNA)-mediated knockdown of Mcl-1, cells were seeded in 6-well plates at 2 × 10^5^ cells per well and grown overnight to approximately 60–70% confluency. Cells were then transfected with 100 nM SignalSilence^®^ Mcl-1-specific siRNA (Cell Signaling Technology, Danvers, MA, USA) or with 100 nM SignalSilence^®^ non-targeting control siRNA, using Lipofectamine RNAiMAX reagent (Thermo Fisher Scientific, Waltham, MA, USA), according to the manufacturer’s instructions. Knockdown efficiency was confirmed 48 hours post-transfection by Western blot analysis using an anti-Mcl-1 rabbit monoclonal antibody (D35A5, Cell Signaling Technology), followed by IRDye800CW conjugated anti-rabbit IgG secondary antibody (Rockland Immunochemicals, Pottstown, PA, USA). Protein bands were visualized using an Odyssey Infrared Imaging System (Li-COR Biosciences, Lincoln, NE, USA), and knockdown efficiency was quantified by densitometry analysis using ImageJ software (NIH, Bethesda, MD, USA).

For RSV infection studies, human alveolar epithelial (A549) cells, treated with either Mcl-1-specific or control siRNA were infected at 48 hours post-transfection with RSV strain VR-1540 (subtype A2) at multiplicities of infection (MOIs) of 1.5 (high inoculum) or 0.5 (low inoculum), performed in triplicate for each condition. After a 1-hour adsorption at 37 °C, viral inoculum was removed, and cells were washed once with phosphate-buffered saline (PBS) before adding fresh DMEM with 2% FBS. Supernatants were harvested at 24 hours post-infection (hpi), and viral titers were quantified by plaque assay on HeLa cell monolayers, as previously described. Statistical analysis was performed using GraphPad Prism software (GraphPad Software, La Jolla, CA, USA). Viral titer differences were analyzed using Student’s t-test, with a p-value of <0.05 considered statistically significant. Statistical Analysis was performed on the data derived from at least three independent experiments, each with triplicate samples.

For influenza virus studies, human alveolar epithelial A549 cells were transfected with either Mcl-1-specific siRNA or non-targeting control siRNA. At 48 hours post-transfection, cells were infected with influenza A/PR/8/34 (H1N1) at MOI 1, and supernatants were collected at 24 hours post-infection. Viral titers were quantified by plaque assay on MDCK cells. Statistical analysis was performed as described above for RSV infection studies.

### Statistical analysis

All data are presented as mean ± standard deviation (SD) unless otherwise noted, based on at least three independent experiments, each with triplicate samples. Statistical analyses were performed using GraphPad Prism 6.01 (GraphPad Software). For comparisons between two groups, a Student’s *t*-test was used. For comparisons among more than two groups, one-way analysis of variance (ANOVA) was performed, followed by Tukey’s multiple comparisons test to assess pairwise differences. A *p* value < 0.05 was considered statistically significant.

## Results

### The cDNA microarray analysis revealed that RSV infection triggers Mcl-1 upregulation.

To elucidate host-virus interactions at the transcriptional level, we analyzed differential gene expression in HEp-2 cells infected with RSV using cDNA microarray analysis complemented by quantitative PCR (qPCR) (**Figure 1**). HEp-2 cells were infected with RSV at a multiplicity of infection of 1, and total RNA was isolated 24 hours post-infection for gene expression profiling. Radiolabeled cDNA probes from RSV-infected and mock-infected cells were hybridized to Atlas^®^ Human Cancer cDNA arrays, and differential gene expression was quantified by densitometry. We identified 12 host genes that were significantly upregulated in RSV-infected cells from the cDNA microarray (≥2-fold increase, *P*<0.01), confirmed by qPCR, encompassing functional categories including cell cycle regulation, cytoskeletal organization, apoptosis modulation, immune evasion, and inflammation. Among the most notable changes, anti-apoptotic Mcl-1 was elevated ~32-fold, while pro-apoptotic caspase-4 showed a modest 1.6-fold increase, suggesting a complex modulation of cell death pathways. GADD153/CHOP, a transcription factor activated by ER stress, was also strongly induced (~10-fold). Unexpectedly, Ubiquitin C (UBC) expression rose >32-fold, indicating a robust activation of the ubiquitin-proteasome system. The most striking increase was observed for interleukin-6 (IL-6), which was elevated ~128-fold. Given IL-6’s role in inflammation and immune regulation, this finding aligns with clinical reports linking high IL-6 levels to severe RSV disease and poor respiratory outcomes in infants.

### Mcl-1 knockout enhances RSV replication in MEF cells

**Figure 2:**
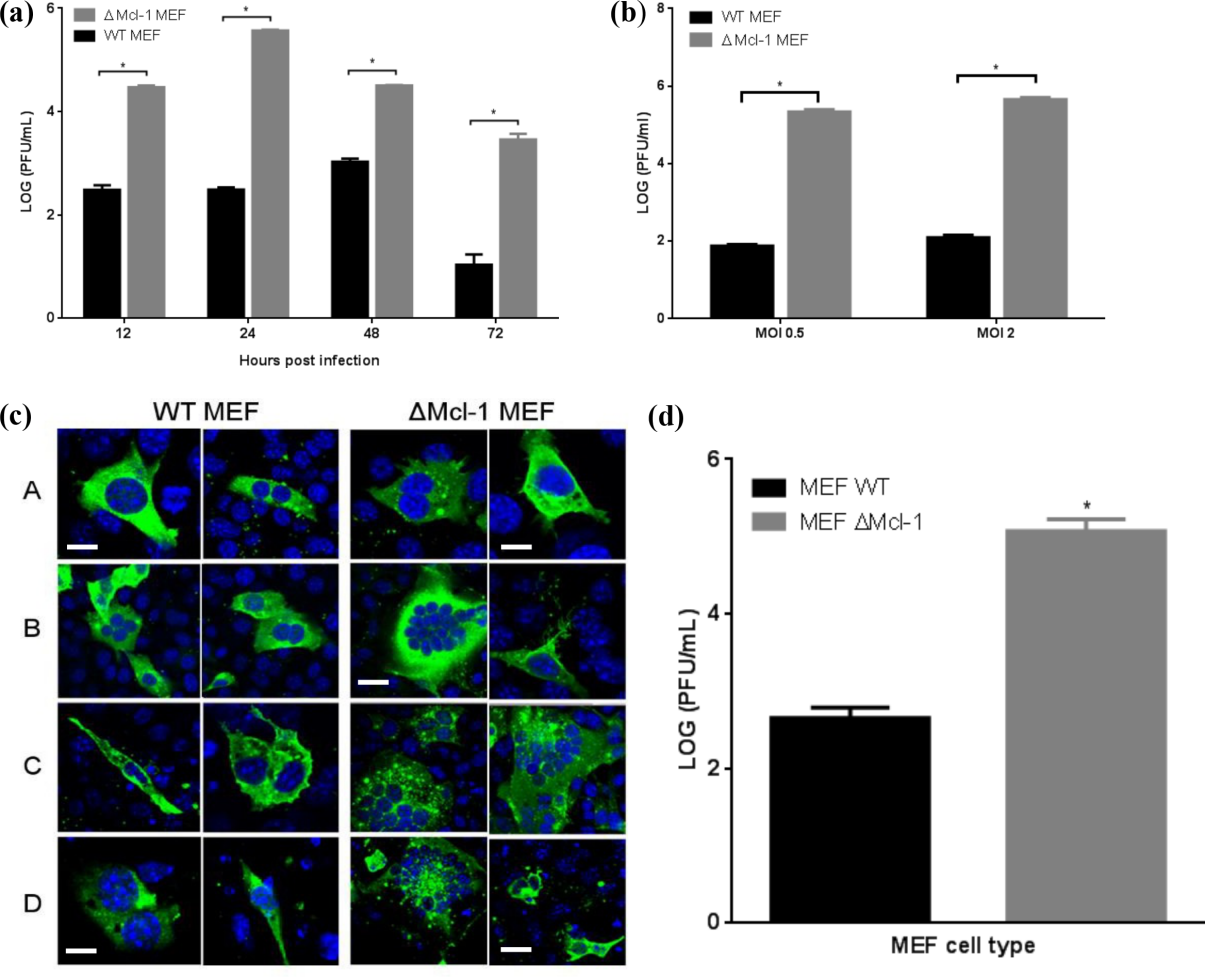
**(A)** Time series of RSV replication in WT and ΔMcl-1 MEF cells. WT and ΔMcl-1 MEF cells were infected with RSV at an MOI of 2. Replication of RSV was measured after 12, 24, 48, and 72 hours post infection for each of the MEF cell lines. Viral titers were quantified by plaque assay and reported as the log (PFU/ml). Error bars represent standard deviation (SD) and significant differences indicated by asterisks (p < 0.001, ANOVA with Tukey post-test. **(B)** RSV replication in MEF cells. WT and ΔMcl-1 MEF cells were infected with RSV at both a high MOI of 2 and a low MOI of 0.5. RSV titers were measured by plaque assay from cell supernatants 24 hours post infection. Error bars indicate standard deviation (SD) and asterisks indicate significant differences in replication between MEF cell types (p < 0.001, ANOVA with Tukey post-test). **(C)** Confocal microscopy time series of RSV infection in MEF cells. At appropriate time points (12, 24, 48, and 72 hpi), MEF cells were prepared for confocal imaging; cells were stained with primary goat polyclonal anti-RSV antibody and then secondary AlexaFluor 488 rabbit anti-goat antibody. Images were acquired via confocal microscopy with a Leica Zeiss LSM510 META with Axiovert 200 motorized microscope and LSM software. Displayed are two representative micrographs each for both WT and ΔMcl-1 MEF cells infected with RSV at an MOI of 2. Horizontal rows represent hours post infection (hpi) when cells were fixed and prepared for microscopy A: 12 hpi; B: 24hpi; C: 48 hpi; D: 72 hpi. Cells were stained with DAPI (blue) and anti- RSV polyclonal antibody (green). All images were captured using the same 63× objective under identical acquisition settings. Bar represents 10 μm. **(D)** Influenza virus replication in MEF cells. WT and ΔMcl-1 MEF cells were infected with influenza virus A/PR/8/34 (H1N1) at an MOI of 2. Influenza titers were measured by plaque assay from cell supernatants 72 hours post infection. Error bars indicate standard deviation (SD) and the asterisk indicates a significant difference in replication between MEF cell types (p < 0.001, Student’s *t* test.

We first compared RSV replication in wild-type (WT) versus Mcl-1 knockout MEFs (ΔMcl-1 MEFs). WT and ΔMcl-1 MEF cells were infected with RSV at MOI 2, and viral titers in the supernatants were measured at 12, 24, 48, and 72 hpi. **Figure 2A** shows the RSV growth curves for each cell type. At every time point, ΔMcl-1 MEFs produced significantly higher virus titers than WT MEFs (*p* < 0.001 at 12, 24, 48, and 72 hpi). On average, the ΔMcl-1 cells yielded over 10-fold (1 log_10_) more PFU than WT cells at each time, with the largest difference observed at 24 hpi (over a 1000-fold, or >3 log_10_, increase in ΔMcl-1 vs. WT). Further, RSV-infected wild-type (WT) and Mcl-1 knockout (ΔMcl-1) MEF cells were harvested at 24 hpi to assess whether the increased viral titers observed in ΔMcl-1 cells were due to enhanced viral replication or simply increased release. Intracellular viral titers were quantified by plaque assay from lysates of infected cells and RSV genome copies were measured by RT-qPCR targeting the N gene in parallel cell lysates. Both assays revealed significantly higher intracellular viral levels in ΔMcl-1 MEFs compared to WT MEFs, indicating that the elevated extracellular titers observed in earlier experiments reflect true enhancement of replication rather than viral egress alone (**Supplementary Figure S1**). These results indicate that the absence of Mcl-1 – an anti-apoptotic protein – allows greater RSV replication, making cells more permissive to infection.

### Increased RSV replication in Mcl-1 knockout MEFs (ΔMcl-1 MEFs) is independent of the initial MOI

To determine whether the enhanced RSV propagation in ΔMcl-1 cells was influenced by the amount of virus used to infect the cells, we performed infections at two different MOIs. WT and ΔMcl-1 MEFs were infected with RSV at a low MOI (0.5) or a high MOI (2), and viral titers were measured at 24 hpi (**Figure 2B**). ΔMcl-1 MEFs again produced significantly higher titers than WT at both MOI 0.5 and MOI 2 (*p* < 0.001 for each comparison). As expected, increasing the MOI from 0.5 to 2 raised the absolute titers in both cell types (approximately 1.5-fold higher on average for each). Importantly, the magnitude of the difference between WT and ΔMcl-1 cells remained large at both inoculum doses. Thus, the ability of RSV to replicate more efficiently in Mcl-1–knockout cells is not simply due to differences in initial viral input; rather, it is an inherent property of the cells lacking Mcl-1.

### Confocal microscopy reveals enhanced syncytium formation in RSV-infected Mcl-1 knockout MEFs

We next examined whether the absence of Mcl-1 altered the cytopathic effect or cell-to-cell spread of RSV infection. WT and ΔMcl-1 MEFs infected with RSV (MOI 2) were fixed at 12, 24, 48, and 72 hpi and analyzed by immunofluorescence confocal microscopy (**Figure 2C**). At 12 hpi, both WT and ΔMcl-1 cultures showed infected cells (green staining) with occasional small syncytia (multi-nucleated cell clusters). By 24 hpi, striking differences emerged: ΔMcl-1 MEFs displayed numerous large syncytia, encompassing many nuclei, whereas WT MEFs had fewer and smaller syncytia. This trend continued at 48 hpi—ΔMcl-1 cultures had abundant, oversized syncytia, while WT cultures showed only the typical moderate syncytia. By 72 hpi, overall cell numbers had decreased in both cultures due to RSV-induced cytopathicity, but the ΔMcl-1 cells still showed markedly larger syncytia than WT. These observations suggest that loss of Mcl-1 leads to more extensive cell fusion during RSV infection, which may facilitate the observed increase in viral spread and titer.

### Mcl-1 knockout also increases influenza virus replication in MEFs

To determine if the effect of Mcl-1 knockout on viral replication extends to other viruses, we tested influenza A virus in the MEF system. WT and ΔMcl-1 MEFs were infected with influenza A/PR/8/34 (H1N1) at MOI 2, and viral titers were measured at 72 hpi. We found that influenza virus also replicated to much higher titers in ΔMcl-1 MEFs than in WT MEFs. As shown in **Figure 2D**, the mean influenza titer in ΔMcl-1 cells was over 2 log_10_ greater than in WT cells (*p* < 0.001). This demonstrates that the phenomenon of increased virus production in the absence of Mcl-1 is not unique to RSV; Mcl-1 knockout similarly boosts replication of influenza virus, indicating a broader antiviral role for Mcl-1.

### Western blot confirms lack of Mcl-1 in Mcl-1 knockout MEFs

**Figure 3:**
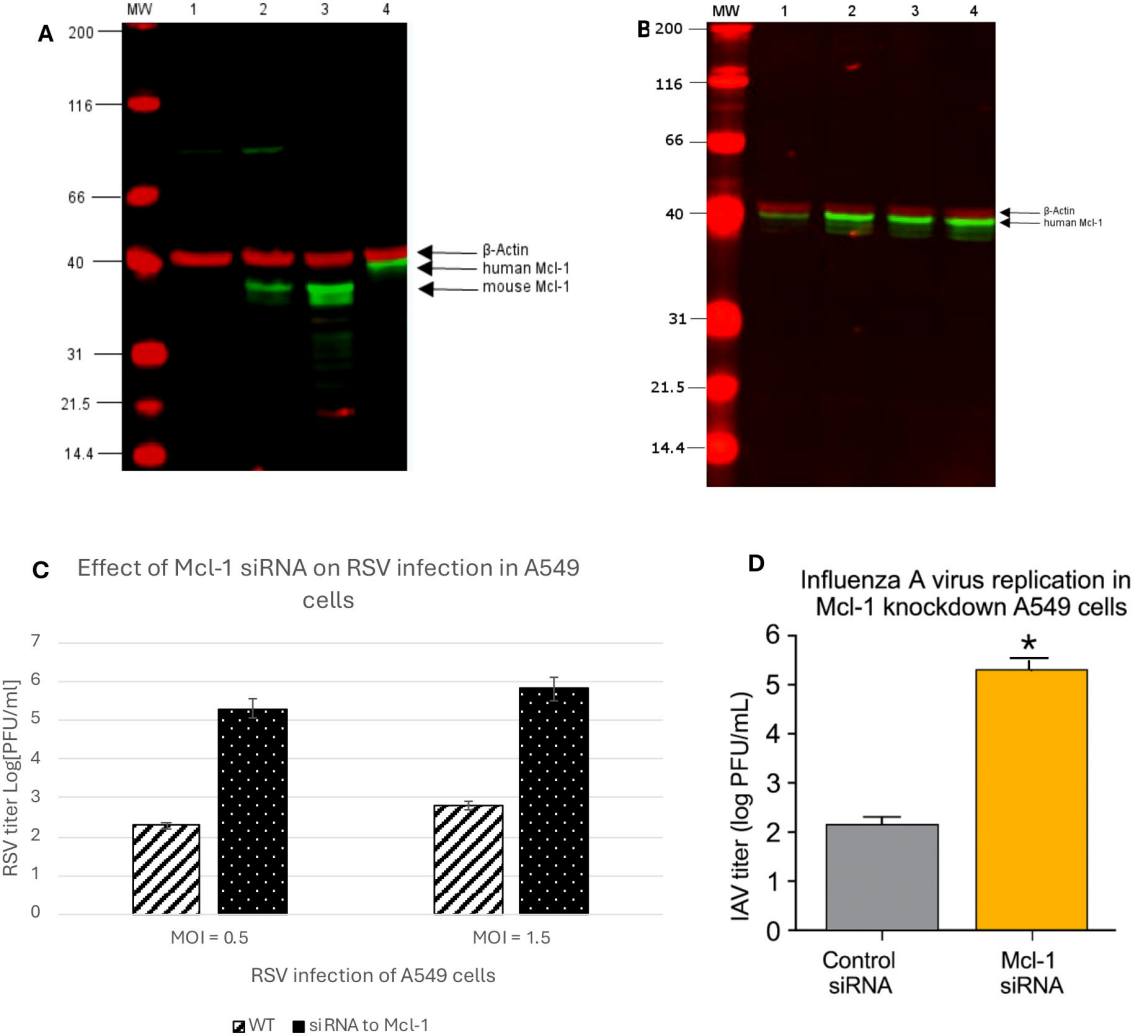
**(A)** Detection of Mcl-1 protein in cell lysates by western blot. Lane MW: Prestained SDS-PAGE Standards; lane 1: ΔMcl-1 MEF cell lysate; lane 2: WT MEF cell lysate; lane 3: RAW264.7 macrophages cell lysate; lane 4: A549 cell lysate. 800 nm channel indicates Mcl-1 protein expression in cell lysates detected by primary rabbit Mcl-1 (D35A5) antibody (Cell signaling, Danvers, MA, USA) and IRDye800 conjugated Rabbit IgG (Rockland, Pottstown, PA, USA) secondary antibody. The 700 nm channel detects the expression of β-Actin as a lysate control by primary mouse β-Actin antibody (Santa Cruz Biotechnology, Dallas TX) and secondary IRDye700 conjugated mouse antibody (Rockland). **(B)** Detection of Mcl-1 protein expression in siRNA treated A549 cells by western blot. A549 cell lysates were analyzed for Mcl-1 expression to confirm knockdown of Mcl-1 using siRNA specific to human Mcl-1 (Cell Signaling, Danvers, MA, USA). A control-siRNA and a transfection control cell lysate were collected as treatment controls and a media mock treatment as a baseline control for Mcl-1 expression in A549 cells. Lane MW: Prestained SDS-PAGE Standards; lane 1: Mcl-1-siRNA A549 cell lysate; lane 2: control- siRNA A549 cell lysate; lane 3: transfection control A549 cell lysate; lane 4: A549 cell lysate. The 800 nm channel indicates Mcl-1 protein expression in cell lysates detected by primary rabbit Mcl-1 (D35A5) antibody (Cell Signaling) and IRDye800 conjugated Rabbit IgG (Rockland, Pottstown, PA, USA) secondary antibody. The 700 nm channel detects the expression of β- Actin as a lysate control by primary mouse β-Actin antibody (Santa Cruz Biotechnology, Dallas TX, USA) and secondary IRDye700 conjugated mouse antibody (Rockland). **(C)** RSV replication in Mcl-1 knockdown A549 cells. Human alveolar epithelial A549 cells were transfected with 100 nM SignalSilence^®^ Control siRNA or SignalSilence^®^ Mcl-1 siRNA followed by infection with RSV at both a high MOI of 1.5 and a low MOI of 0.5. RSV titers were measured by plaque assay from cell supernatants 24 hours post infection. Data represent mean ± SD from three independent experiments. *p* < 0.001 (Student’s t-test). **(D)** Influenza A virus replication in Mcl-1 knockdown A549 cells. Human alveolar epithelial A549 cells were transfected with either Mcl-1-specific siRNA or non-targeting control siRNA. At 48 hours post-transfection, cells were infected with influenza A/PR/8/34 (H1N1) at MOI 1, and supernatants were collected at 24 hours post-infection. Viral titers were quantified by plaque assay on MDCK cells. Data represent mean ± SD from three independent experiments. *p* < 0.001 (Student’s t-test).

The finding that ΔMcl-1 MEFs support much higher RSV replication than WT was unexpected. Although the ΔMcl-1 mutation in these MEFs was previously established (Opferman et al., 2003), we confirmed the absence of Mcl-1 protein in our cells and compared Mcl-1 expression across cell types relevant to RSV. Lysates from WT MEFs, ΔMcl-1 MEFs, human alveolar epithelial (A549) cells, and murine RAW 264.7 cells were subjected to Western blot analysis (with an antibody detecting both mouse and human Mcl-1) alongside a β-actin loading control. As shown in **Figure 3A**, WT MEFs displayed a clear Mcl-1 band at ~35 kDa (mouse Mcl-1), whereas ΔMcl-1 MEFs showed no detectable Mcl-1 protein. A549 cell lysates yielded an Mcl-1 band at ~40 kDa (the expected size for human Mcl-1), and RAW264.7 macrophages again showed a ~35 kDa band (mouse Mcl-1). The β-actin bands (~42 kDa) were equally present in all samples, confirming equal protein loading. This reconfirms that the ΔMcl-1 MEF line completely lacks Mcl-1, and thus the enhanced RSV replication observed is attributable to the loss of Mcl-1.

### Mcl-1 knockdown in human alveolar epithelial (A549) cells increases RSV and influenza replication (implications for cancer therapy)

Given that Mcl-1 is often targeted in cancer therapy, we examined whether reducing Mcl-1 in human cells would have a similar effect on RSV and influenza. Human type II alveolar epithelial A549 cells were transfected with Mcl-1-specific siRNA to knock down Mcl-1, or with a non-targeting control siRNA. Western blot analysis confirmed that Mcl-1 protein levels were dramatically reduced in Mcl-1 siRNA-treated A549 cells compared to controls (**Figure 3B**). We then infected these cells with RSV at two different inocula (MOI 1.5 for a relatively high dose, and MOI 0.5 for a lower dose) and measured viral titers at 24 hpi. RSV replicated to much higher levels in Mcl-1–depleted cells than in control cells under both conditions. As shown in **Figure 3C**, knockdown of Mcl-1 led to RSV titers more than 3 log_10_ greater than those in control siRNA-treated cells (*p* < 0.001). Similarly, Mcl-1 knockdown resulted in a ~3 log_10_ PFU/mL increase at 24 hpi in influenza virus titers compared to control cells (**Figure 3D**), indicating that Mcl-1 may exert a broader antiviral effect beyond RSV. This aligns with prior reports indicating low apoptosis induction by influenza virus in A549 cells (Kotelkin et al., 2003), suggesting that Mcl-1’s antiviral function may involve mechanisms beyond apoptotic regulation. Although Mcl-1 knockdown enhances replication of both RSV and IAV, RSV appears more sensitive to Mcl-1 depletion. These experiments suggest important clinical implications regarding the use of Mcl-1 inhibitors in cancer therapy. Patients undergoing treatments targeting Mcl-1 could be more susceptible to severe RSV and influenza infection or other respiratory pathogens, due to the virus being able to replicate uncontrolled when Mcl-1 is suppressed. Therefore, clinical strategies should consider proactive viral monitoring or prophylactic antiviral measures for patients receiving Mcl-1 inhibitor treatments.

### Effects of apoptosis induction or inhibition on RSV replication in MEFs

**Figure 4:**
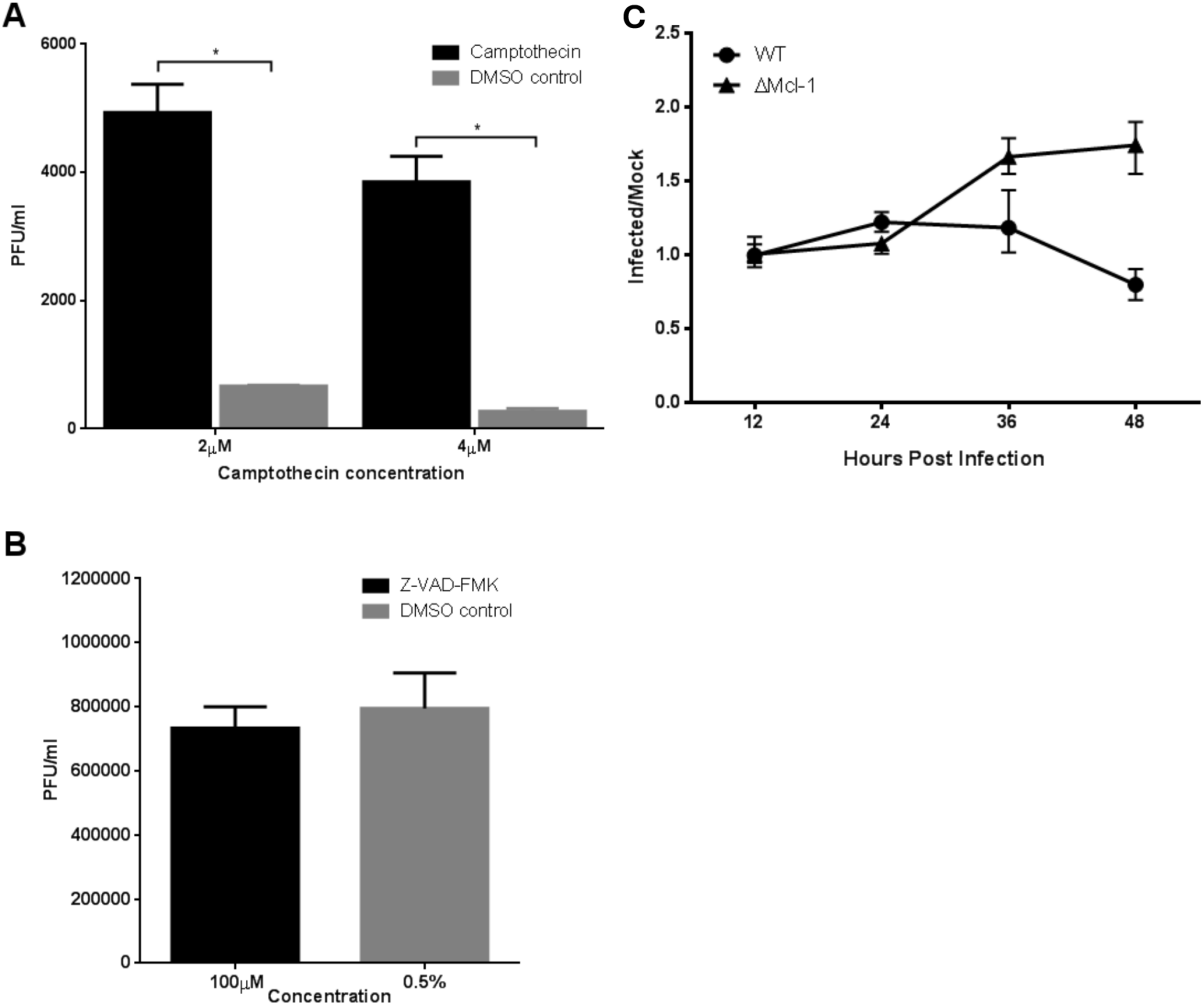
**(A, B)** RSV replication in MEF cells treated with apoptosis inducing or inhibiting compounds. WT MEF cells were treated with apoptosis inducing camptothecin (CPT) while ΔMcl-1 MEF cells received Z-VAD-FMK, an apoptosis inhibitor. RSV replication was measured by titration of cell culture media 24 hours post infection by plaque assay. Error bars represent standard deviation (SD) and asterisks indicate significant differences (p < 0.001, ANOVA with Tukey post-test). **(A)** Replication of RSV in WT MEF cells treated with 2 µM or 4 µM CPT to induce apoptosis. DMSO was added to untreated cells at the concentrations used in treated wells to control for the DMSO used as a solvent for CPT. **(B)** Replication of RSV in ΔMcl-1 MEF cells treated with 100 µM Z-VAD-FMK. DMSO was added to otherwise untreated cells to control for the concentration added by treatment with Z-VAD-FMK. **(C)** Time series of apoptosis activity in RSV infected MEF cells measured by the Apo-ONE Homogeneous Caspase-3/7 Assay. Caspase 3/7 cleavage activity was measured in both WT and ΔMcl-1 MEF cells as fluorescence at 485 nm/535 nm (excitation/emission) in triplicate wells. Apoptosis was monitored for a total of 48 hours with absorbance reading taken every 12 hours. The ratio of caspase 3/7 activity in RSV infected cells to control mock infected cells is plotted to control for background differences in caspase levels between cell types. Error bars represent 95% confidence intervals.

To further probe the role of apoptosis in the differential viral replication, we experimentally manipulated apoptosis in WT and ΔMcl-1 MEFs during RSV infection. We induced apoptosis in WT MEFs using Camptothecin (CPT) and inhibited apoptosis in ΔMcl-1 MEFs using the pan-caspase inhibitor Z-VAD-FMK, then measured RSV titers. In WT MEFs, forcing apoptosis with CPT significantly increased RSV replication at 24 hpi (**Figure 4A**). Specifically, CPT-treated WT cells produced over seven times more virus than DMSO-treated control cells (*p* < 0.001). Interestingly, WT cells treated with 2 µM CPT yielded higher titers than those treated with 4 µM CPT, likely because the higher DMSO content (0.2% vs 0.1%) at 4 µM had additional cytotoxic effects that partially countered the benefit of apoptosis induction. In ΔMcl-1 MEFs, on the other hand, blocking apoptosis with 100 µM Z-VAD-FMK had no significant effect on RSV titers compared to the DMSO-treated ΔMcl-1 controls at 24 hpi (**Figure 4B**) and 48 hpi (**Supplementary Figure S2**). At both 24 and 48 hpi, despite suppression of caspase-3/7 activity by Z-VAD-FMK, RSV titers in ΔMcl-1 MEFs remained elevated, confirming that enhanced replication is not dependent on apoptosis at late timepoints (48 hpi) either. Taken together, these experiments indicate that while enhanced apoptosis can make WT cells somewhat more permissive to RSV (as seen with CPT treatment), the extreme permissiveness of ΔMcl-1 cells cannot be reversed by simply inhibiting apoptosis. In other words, the increased RSV replication in ΔMcl-1 cells is only partially attributable to apoptotic differences, implying that Mcl-1’s influence on RSV infection involves additional factors beyond apoptosis regulation.

### Mcl-1 knockout MEFs do not upregulate apoptosis until late in RSV infection

Mcl-1’s primary known function is to inhibit apoptosis by antagonizing pro-apoptotic proteins. We therefore investigated the timing of apoptosis in RSV-infected MEFs to see if differences in cell death could explain the enhanced viral replication in ΔMcl-1 cells. We measured caspase-3/7 activity (as an indicator of apoptosis) in WT and ΔMcl-1 MEFs at multiple time points post-RSV infection (every 12 hours up to 48 hours). The fluorescence readings in infected wells were normalized to mock-infected controls to account for basal activity and cell number (see Methods). As shown in **Figure 4C**, no substantial increase in caspase activity was detected in ΔMcl-1 MEFs relative to WT MEFs during the early phase of infection (up to 24 hpi). A noticeable divergence occurred at 36 hpi: ΔMcl-1 MEFs showed a significant rise in caspase-3/7 activity, whereas WT MEFs had a decline in caspase activity at the same time point. By 48 hpi, apoptosis levels in ΔMcl-1 cells remained elevated relative to the earlier time points, whereas WT cells did not show a corresponding increase. These results were somewhat surprising – despite the drastic differences in virus replication apparent by 12–24 hpi, apoptosis in ΔMcl-1 cells was not significantly higher than in WT cells until much later (36–48 hpi). This suggests that early in RSV infection, the absence of Mcl-1 enhances viral replication through a mechanism that does not involve an immediate increase in apoptosis.

## Discussion

RSV is a significant respiratory pathogen worldwide, especially dangerous for young children and the elderly. During viral infections (including RSV), host cells often upregulate Mcl-1 (Lindemans et al., 2006; Zhong et al., 2012). Twelve host genes including Mcl-1 were significantly upregulated in RSV-infected cells based on microarray analysis (≥2-fold, *P* < 0.01), with findings confirmed by qPCR. These genes span key functional categories, including cell cycle control, cytoskeletal organization, apoptosis regulation, immune evasion, and inflammation. Notably, anti-apoptotic **Mcl-1** increased ~32-fold, while pro-apoptotic caspase-4 showed a more modest 1.6-fold rise, indicating a complex regulation of cell death pathways. Our observation of Mcl-1 upregulation in RSV-infected HEp-2 cells aligns with previous studies demonstrating similar upregulation in A549 cells and in primary human bronchial epithelial cells (NHBE and SAEC) (Kotelkin et al., 2003). This indicates that Mcl-1 upregulation during RSV infection is not cell-type restricted. Notably, Rajan et al. (2022) compared transcriptomic responses between HEp-2 and A549 cells and found key differences in antiviral gene expression profiles, emphasizing the importance of validating observations across multiple respiratory epithelial cell models. Another gene in the apoptosis/DNA damage category, **GADD153** (also known as CHOP, a transcription factor activated by ER stress and DNA damage), was elevated ~10-fold. Interestingly, in RSV-infected cells **Ubiquitin C (UBC)** transcript levels were ~>32-fold higher than in controls. This unexpected surge suggests that RSV infection triggers a strong activation of the ubiquitin-proteasome system. **Interleukin-6 (IL-6)**, a multifunctional cytokine, was significantly increased (~128-fold). IL-6 is produced by airway epithelial cells and various immune cells in response to viral infections and tissue damage. In the context of RSV, IL-6 has a dual role: it contributes to the acute inflammatory response (fever, acute phase protein production, leukocyte recruitment) and also has immunoregulatory effects (e.g., promoting B-cell maturation and T-cell activation) (Castell et al., 1989; Maeda et al., 2010). Elevated IL-6 in RSV-infected cells is consistent with clinical observations that IL-6 levels are detectable in respiratory secretions of infants with RSV bronchiolitis (Levitz et al., 2012). High concentrations of IL-6 in the airways have been associated with severe RSV disease, such as those requiring mechanical ventilation (Russell et al., 2017).

In this study, we also show for the first time that knocking out Mcl-1 in MEF cells leads to markedly higher RSV replication compared to WT cells. This heightened permissiveness was evident very early in infection (as soon as 12 hpi in ΔMcl-1 cells). However, the modulation of apoptosis – the canonical function of Mcl-1 – did not correspondingly differ at those early times. In fact, we did not observe a significant increase in apoptosis (caspase-3/7 activity) in ΔMcl-1 MEFs until 36 hpi and later, which is when WT cells actually began to show declining apoptotic activity. Furthermore, artificially inducing apoptosis in WT cells (with CPT) increased RSV replication but inhibiting apoptosis in ΔMcl-1 cells (with Z-VAD-FMK) did not reduce their already high replication. These data suggest that Mcl-1’s effect on RSV replication is not solely through its role in preventing apoptosis, at least in the initial stages of infection. There may be additional, apoptosis-independent mechanisms by which Mcl-1 influences viral replication.

Confocal microscopy provided insight into phenotypic differences during infection: ΔMcl-1 MEFs formed unusually large and numerous syncytia when infected with RSV, in stark contrast to WT cells. The RSV fusion (F) protein is known to drive syncytium formation and also to induce apoptosis at later stages of infection (Harris and Werling, 2003; Eckardt-Michel et al., 2008). We observed that ΔMcl-1 cells had both enhanced syncytia formation and increased late-stage apoptosis. This raises the intriguing possibility that Mcl-1 might interact with or regulate processes initiated by the RSV F protein. In the absence of Mcl-1, cell-cell fusion mediated by F protein could proceed unchecked, leading to more extensive syncytia and facilitating viral spread. It is conceivable that Mcl-1 normally acts to limit the extent of fusion or to mitigate cell damage in some way during RSV infection. Discovering a physical or functional interaction between Mcl-1 and the RSV fusion machinery would be an exciting avenue for future research to explain some of our observations.

To assess whether this effect was conserved across cell types, we knocked down Mcl-1 in human A549 cells, a widely used epithelial model for RSV. The knockdown similarly enhanced RSV titers (**Figure 3C**), supporting the relevance of this antiviral effect in human cells. Additionally, we observed increased influenza A virus replication in ΔMcl-1 MEFs and in Mcl-1-silenced A549 cells (**Figure 3D**), suggesting a broader antiviral role for Mcl-1 across multiple RNA viruses. These results align with Kotelkin et al., who showed that Mcl-1 is upregulated in A549 and NHBE cells during RSV infection, but extend their findings by functionally linking Mcl-1 to viral restriction.

Mcl-1 is somewhat unique among anti-apoptotic BCL-2 family members in that it has a very short half-life and has been implicated in multiple cellular processes beyond apoptosis, including mitochondrial dynamics and cell metabolism (Perciavalle et al., 2012; Rinkenberger et al., 2000; Wang et al., 2014; Fleischer et al., 2006; Gui et al., 2015). Our findings align with a prior report that small interfering RNA (siRNA)-mediated depletion of Mcl-1 did not significantly affect apoptosis under certain conditions (Minet et al., 2006). This suggests that Mcl-1 could influence virus replication through a novel function or pathway that does not directly involve the classic caspase-dependent apoptosis cascade. For example, Mcl-1 might affect viral replication by modulating autophagy, innate immune signaling, or some aspect of cellular physiology that viruses exploit. The precise mechanism remains to be determined, but the key point is that the role of Mcl-1 in RSV infection appears to extend beyond simply keeping cells alive.

An important implication of our work is that Mcl-1 upregulation during RSV infection likely reflects a host antiviral defense rather than a proviral strategy. Although one might expect a virus to benefit from inhibiting apoptosis by inducing Mcl-1, our findings indicate the opposite—RSV replication increases in the absence of Mcl-1. Thus, Mcl-1 may be induced early in infection as part of the host’s effort to limit viral propagation through apoptosis or other antiviral mechanisms.

Interestingly, manipulating Mcl-1 could have practical applications in vaccine production, where maximizing virus yield is often beneficial. Our findings show that inhibiting Mcl-1 enhances replication of both RSV and influenza A virus in cell culture. This approach could be combined with other yield-boosting strategies, such as interferon suppression using antisense morpholino oligomers (Prescott et al., 2024), to further improve vaccine virus production. However, any such strategy would require careful consideration of cell viability and production efficiency.

On the other hand, our results raise a caution for clinical contexts: Mcl-1 is a prominent target in cancer therapy (Glaser et al., 2012; Deng et al., 2024) because many tumors rely on Mcl-1 for survival and chemoresistance. Several Mcl-1 inhibitors are in development or clinical trials for treating malignancies. Our data indicate that systemic inhibition or knockdown of Mcl-1 can strongly amplify virus replication (as demonstrated in A549 cells for RSV and influenza virus). Immunocompromised cancer patients already face high risks for infections; if treated with an Mcl-1 inhibitor, they might become even more susceptible to severe viral infections due to this “permissive” effect on viruses. This phenomenon should be investigated further in animal models and clinical settings. It will be important to monitor viral infection rates or severity in patients receiving Mcl-1-targeting drugs and to ensure appropriate prophylactic measures (such as vaccinations or antiviral therapies) are in place.

In conclusion, our work reveals a novel facet of the interaction between RSV or influenza virus and the host apoptosis regulator Mcl-1. The absence of Mcl-1 greatly enhances RSV (and influenza) replication, indicating that Mcl-1 normally acts to restrict these viruses. This restriction is only partially explained by apoptosis modulation, suggesting Mcl-1 has additional antiviral mechanisms. A deeper understanding of Mcl-1’s role in RSV and other viral infections will improve our knowledge of viral pathogenesis and could inform the development of therapies that carefully consider the balance between controlling cancer and maintaining antiviral defenses. Exploiting Mcl-1’s antiviral function might also provide new strategies to combat RSV, a pathogen for which effective treatments and vaccines are still being sought.

## Data Availability

The original contributions presented in the study are included in the article/supplementary material, further inquiries can be directed to the corresponding author/s.

## Supplementary material

Supplementary Figure 1 Mcl-1 knockout increases RSV replication and infectious virus production. (A) Intracellular RSV titers (PFU/mL, plaque assay) and RSV N gene copy numbers (RT-qPCR) from WT and ΔMcl-1 MEFs at 24 hours post-infection. Both metrics indicate enhanced viral replication in Mcl-1–deficient cells. (B) Plaque counts from equal supernatant volumes applied to HeLa cells, revealing significantly more infectious virions produced by ΔMcl-1 MEFs. Data represent means ± SD from three independent experiments. *p* < 0.001 for all comparisons (Student’s t-test).

Supplementary Figure 2 Effect of Z-VAD-FMK on RSV replication in ΔMcl-1 MEFs at 48 hours post-infection. ΔMcl-1 MEFs were infected with RSV at an MOI of 2 and treated with either the pan-caspase inhibitor Z-VAD-FMK (100 µM) or vehicle control (0.5% DMSO). Supernatants were collected at 48 hpi, and viral titers were determined by plaque assay. Data represent mean ± SD from three independent experiments. *p* > 0.05 (Student’s t-test).
