# Supplementary material for: Myeloid Cell Leukemia-1 knockout leads to increased viral propagation of Respiratory Syncytial Virus and influenza virus in mouse embryonic fibroblast cells and A549 cells: implications in cancer therapy
Source: Front Cell Infect Microbiol. 2025 Aug 29;15:1615790. doi: 10.3389/fcimb.2025.1615790 (PMC12426169; doi:10.3389/fcimb.2025.1615790)
Supplement: Supplementary Figure 1 — Mcl-1 knockout increases RSV replication and infectious virus production. (A) Intracellular RSV titers (PFU/mL, plaque assay) and RSV N gene copy numbers (RT-qPCR) from WT and ΔMcl-1 MEFs at 24 hours post-infection. Both metrics indicate enhanced viral replication in Mcl-1–deficient cells. (B) Plaque counts from equal supernatant volumes applied to HeLa cells, revealing significantly more infectious virions produced by ΔMcl-1 MEFs. Data represent means ± SD from three independent experiments. p < 0.001 for all comparisons (Student’s t-test). [file SupplementaryFile1.pdf]

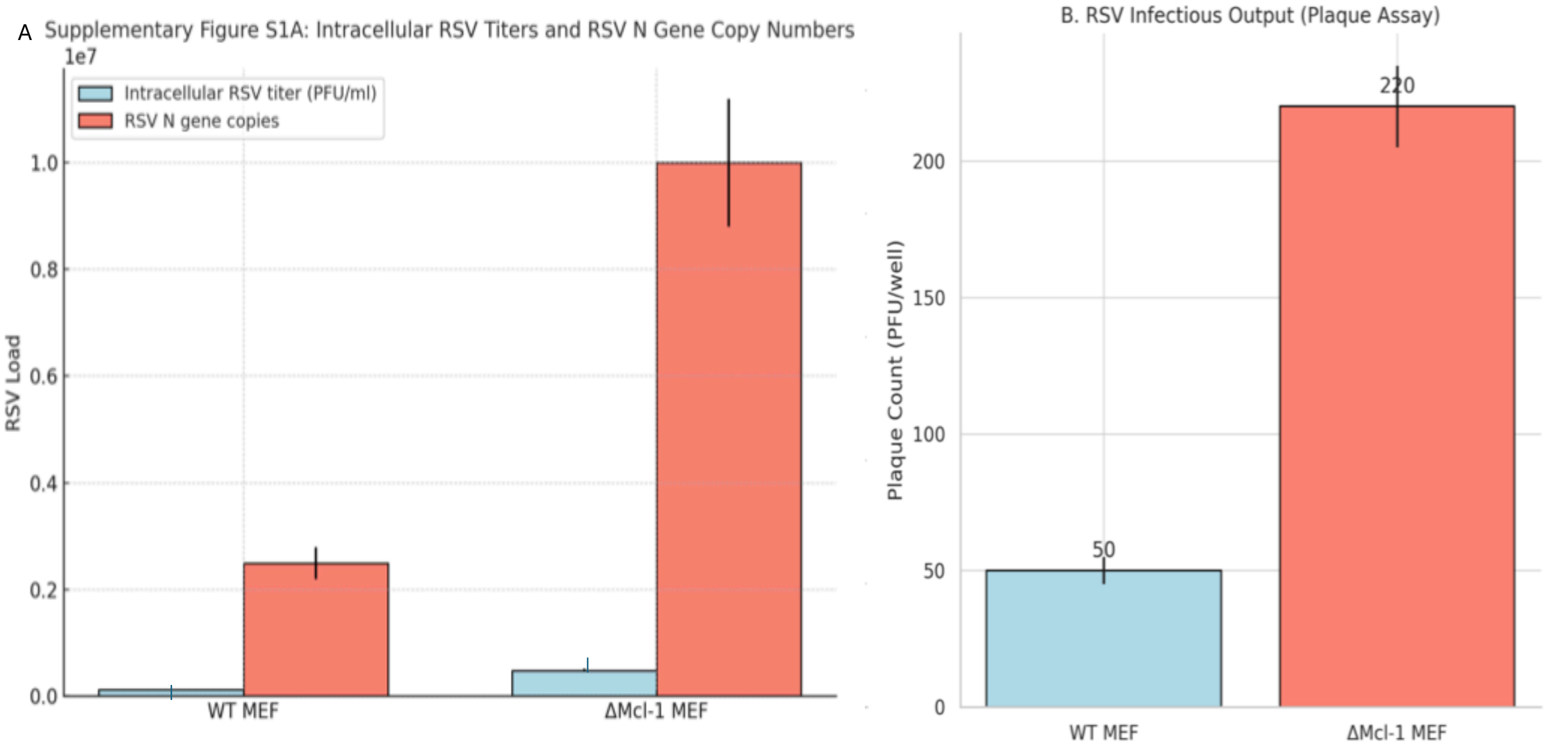

Supplementary Figure S1. Mcl-1 knockout increases RSV replication and infectious virus production.

(A) Intracellular RSV titers (PFU/mL, plaque assay) and RSV N gene copy numbers (RT-qPCR) from WT and ΔMcl-1 MEFs at 24 hours post-infection. Both metrics indicate enhanced viral replication in Mcl-1–deficient cells.

(B) Plaque counts from equal supernatant volumes applied to HeLa cells, revealing significantly more infectious virions produced by ΔMcl-1 MEFs.

Data represent means ± SD from three independent experiments. $p < 0.001$ for all comparisons (Student's t-test).
