# Supplementary material for: Myeloid Cell Leukemia-1 knockout leads to increased viral propagation of Respiratory Syncytial Virus and influenza virus in mouse embryonic fibroblast cells and A549 cells: implications in cancer therapy
Source: Front Cell Infect Microbiol. 2025 Aug 29;15:1615790. doi: 10.3389/fcimb.2025.1615790 (PMC12426169; doi:10.3389/fcimb.2025.1615790)
Supplement: Supplementary Figure 2 — Effect of Z-VAD-FMK on RSV replication in ΔMcl-1 MEFs at 48 hours post-infection. ΔMcl-1 MEFs were infected with RSV at an MOI of 2 and treated with either the pan-caspase inhibitor Z-VAD-FMK (100 µM) or vehicle control (0.5% DMSO). Supernatants were collected at 48 hpi, and viral titers were determined by plaque assay. Data represent mean ± SD from three independent experiments. p > 0.05 (Student’s t-test). [file SupplementaryFile2.pdf]

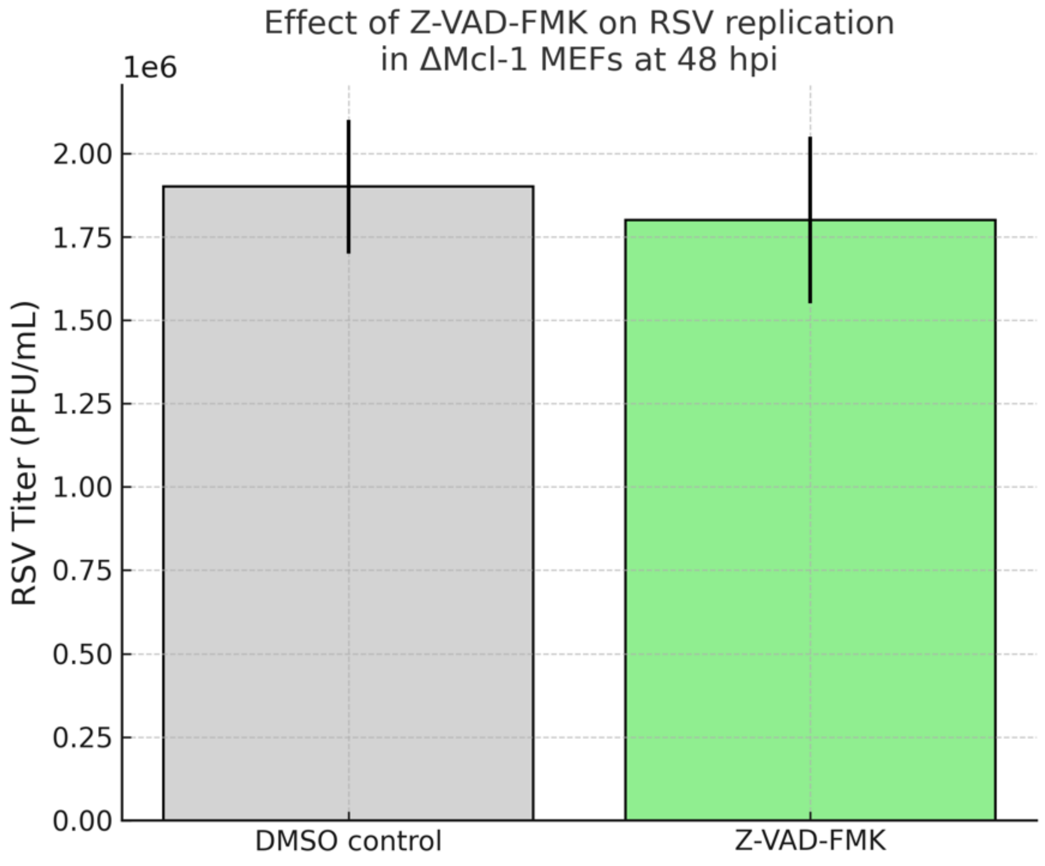

Supplementary Figure S2. Effect of Z-VAD-FMK on RSV replication in ΔMcl-1 MEFs at 48 hours post-infection.

ΔMcl-1 MEFs were infected with RSV at an MOI of 2 and treated with either the pan-caspase inhibitor Z-VAD-FMK (100 μM) or vehicle control (0.5% DMSO). Supernatants were collected at 48 hpi, and viral titers were determined by plaque assay. Data represent mean ± SD from three independent experiments. $p > 0.05$ (Student's t-test).
